# Supplementary material for: Large-scale electron microscopy database for human type 1 diabetes
Source: Nat Commun. 2020 May 18;11:2475. doi: 10.1038/s41467-020-16287-5 (PMC7235089; doi:10.1038/s41467-020-16287-5)
Supplement: Supplementary file 1 — Supplementary Information [file 41467_2020_16287_MOESM1_ESM.pdf]

Extended data

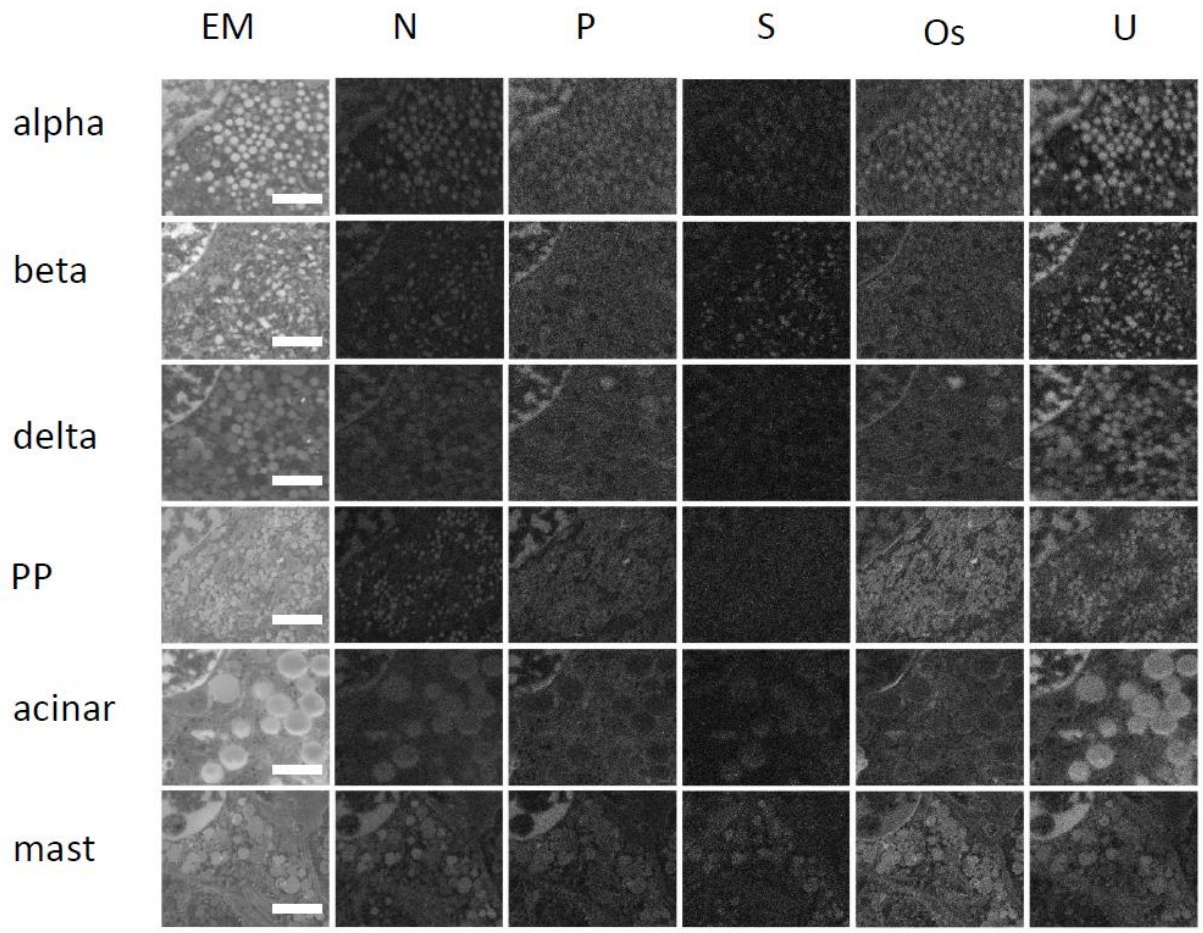

Extended figure 1 | Raw EDX data from different cell types. Elemental specific images displaying nitrogen (N), phosphorus (P), sulfur (S), osmium (Os), and uranium (U) are shown. Micrographs are representative and similar results can be obtained from each dataset. See figure 1c for further details. Bars: 2 µm, indicated in EM micrographs and identical for elemental maps.

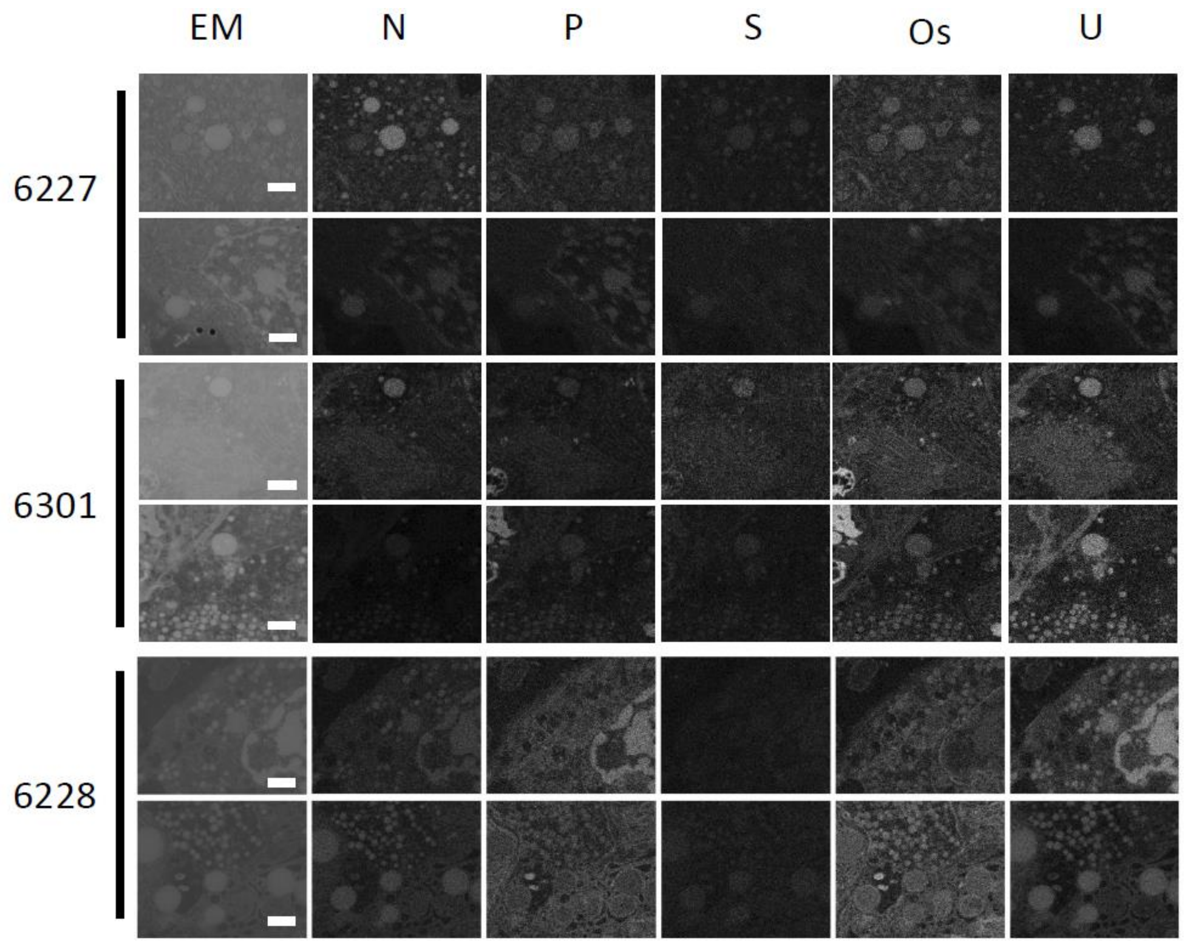

Extended figure 2 | Raw EDX data from intermediate cells. Element specific images displaying nitrogen (N), phosphorus (P), sulfur (S), osmium (Os), and uranium (U) are shown. Data is representative and similar to other donors with intermediate cells for the respective donor groups. See figure 3 for further details. Bars: 1 µm, indicated in EM micrographs and identical for elemental maps.

Supplementary information

Table S1 | nPOD donor information. Donor Case-IDs are indicated and data is sorted by age of demise. Information about donors are on pancreas donor region, disease duration, gender, race, body mass index (BMI), serum HbA1c (mM), serum C-peptide (ng/ml), type of autoantibodies present measured by radioimmunoassay (RIA), and human leukocyte antigen (HLA) genotype per donor. Missing data did not pass quality check or is not available.

Condition	Case-ID	Age Demise	Region	Duration	Gender	Race	BMI	HbA1c	C peptide	Autoantibody (RIA)	HLA
Control	6130	5,2	Head		Male	Caucasian	18,5		4,80	Negative	A*01/03, B*07/08, DR*15/17, DQ*02/06
	6232	14,0	Head		Female	Caucasian	20,8		19,50	Negative	A*02/24, B*07/44, DR*15/15, DQ*06/06
	6233	14,0	Head		Male	Caucasian	21,9	5,5	7,26	Negative	A*01/26, B*38/51, DR*01/13, DQ*na/na
	6374	14,0	Head		Female	Caucasian	18,9	6,1	13,42	Negative	A*01/02, B*60/55, DR*13/, DQ*06/
	6153	15,2	Head		Male	Hispanic/Latino	20,5	5,5	8,38	Negative	A*24/26, B*35/38, DR*04/13, DQ*06/08
	6230	16,0	Body		Male	Caucasian	19,4	5,3	5,22	Negative	A*23/32, B*44/49, DR*04/11, DQ*07/08
	6227	17,0	Head		Female	Caucasian	26,4		2,75	Negative	A*02/03, B*60/62, DR*04/13, DQ*06/08
	6412	17,6	Head		Female	Caucasian	24	6,1	45,57	Negative	A*01/11 B*35/62 DR*04/11 DQ*07/08
	6098	17,8	Head		Male	Caucasian	22,8	4,9	1,41	Negative	A*01/32, B*08/27, DR*17/08, DQ*02/04
	6417	18,2	Head/Body		Female	Caucasian	24,5	5,1	3,94	Negative	A*25/31, B*18/60, DR*15/-, DQ*-/-
	6160	22,1	Head		Male	Caucasian	23,9	5,2	0,40	Negative	A*11/29, B*35/44, DR*103/07, DQ*02/05
	6131	24,2	Head		Male	Caucasian	24,8		1,01	Negative	A*01/11, B*08/18, DR*17/17, DQ*02/02
	6126	25,2	Head		Male	Hispanic/Latino	25,1		0,88	Negative	A*/, B*/, DR*/, DQ*/
	6331	27,1	Body		Female	African Am	24	5,4	3,01	Negative	A*26/34, B*35/49, DR*18/15, DQ*04/06
	6229	31,0	Head		Female	Caucasian	26,9	5,5	6,23	Negative	A*/, B*/, DR*/, DQ*/
Pregnancy	6226	38,0	Head		Female	Asian	27,2	5,3	3,87	Negative	A*203/33,B*35/55/DR*11/14,DQ*05/07
AAb+	6421	6,7	Body		Male	Hispanic	17,9	5,6	1,84	GADA+	A*02/03, B*27/62, DR*01/-, DQ*05/-
	6424	17,7	Head/Body		Male	Caucasian	51,4	5,8	6,97	GADA+ mIAA+	A*30/68, B*08/35, DR*17/04, DQ*02/08
	6314	21,0	Head/Body		Male	Caucasian	23,8		1,49	GADA+	A*02/24, B*07/35, DR*103/04, DQ*/
	6397	21,2	Body		Female	Caucasian	29,6	6,0	12,77	GADA+	A*02/- B*51/62 DR*13/15 DQ*-/-
	6197	22,0	Head		Male	African Am	28,2	5,5	17,48	GADA+ IA-2A+	A*02/24, B*42/45, DR*07/18, DQ*02/04
	6303	22,0	Body		Male	Caucasian	31,9	5,4	3,03	GADA+	A*01/11, B*08/44, DR*17/07, DQ*02/02
	6433	24,0	Body		Male	Hispanic	30,8	5,3	4,44	GADA+	A*30/68, B*08/45, DR*04/12, DQ*03/03
	6388	25,2	Body		Female	Hispanic	26	5,7	1,38	GADA+ mIAA+	A*02/, B*35/65, DR*01/04, DQ*05/07
	6400	25,2	Body		Male	Hispanic	22,2	5,5	4,17	GADA+	A*26/31 B*08/62 DR*04/13 DQ*07/08
	6301	26,0	Body		Male	African Am	32,1	5,5	3,92	GADA+	A*23/, B*07/, DR*11/13, DQ*03/06
	6151	30,0	Head		Male	Caucasian	24,2		5,49	GADA+	A*02/24, B*44/44, DR*01/07, DQ*02/05
	6156	40,0	Head		Male	Caucasian	19,8		13,34	GADA+	A*03/32, B*61/53, DR*01/01, DQ*05/05
	6158	40,3	Head		Male	Caucasian	29,7	5,6	0,51	GADA+ mIAA+	A*03/24, B*49/62, DR*04/13, DQ*06/07
T1D	6063	4,4	Head	3,0	Male	Caucasian	23,8	-	<0.05	mIAA+	A*02/24, B*08/62, DR*04/17, DQ*02/08
	6360	4,8	Body	2,5	Female	Caucasian	26,1	10,2	<0,02	mIAA+	A*31/68, B*08/39, DR*04/17, DQ*02/08
	6209	5,0	Head	0,3	Female	Caucasian	15,9		0,10	IA-2A+ ZnT8A+ mIAA+	A* 01:01, 02:01 DRB1* 03:01, 04:01 DQA1* 03:01, 05:01 DQB1 02:01, 03:02
	6380	11,6	Body	0,0	Female	Adrican American	14,6	13,5	0,22	Negative	A*33/68, B*71/53, DR*17/13, DQ*02/06
	6228	13,0	Head/Body	0,0	Male	Caucasian	17,4	13,3	0,10	GADA+ IA-2A+ ZnT8A+	A*23/68, B*60/44, DR*17/04, DQ*02/08
	6243	13,0	Head	5,0	Male	Caucasian	21,3	13,1	0,42	mIAA+	A*01/02, B*08/08, DR*17/17, DQ*02/02
	6113	13,1	Head	1,6	Female	Caucasian	24,8		<0.05	mIAA+	A*02/03, B*08/44, DR*01/17, DQ*02/05
	6087	17,5	Head	4,0	Male	Caucasian	21,9		<0.05	ZnT8A+ mIAA+	A*/, B*/, DR*04/17, DQ*02/08
	6064	19,6	Head	9,0	Female	Caucasian	22,6		<0.05	GADA+ IA-2A+ ZnT8A+ mIAA+	A*02/29, B*44/62, DR*01/04, DQ*/
	6337	20,6	Head	5,0	Female	Caucasian	17,9	12,5	<0,02	mIAA+	A*01/33, B*08/65, DR*01/17, DQ*02/05
	6198	22,0	Tail	3,0	Female	Hispanic/Latino	23,1		<0.05	GADA+ IA-2A+ ZnT8A+ mIAA+	A*02/30, B*18/53, DR*04/11, DQ*/
	6211	24,0	Body	4,0	Female	African Am	24,4	10,5	<0,05	GADA+ IA-2A+ ZnT8A+ mIAA+	A*02/03, B*08/45, DR*04/12, DQ*07/08
	6362	24,9	Body	0,0	Male	Caucasian	28,5	10,0	0,38	GADA+	A*03/11, B*18/35, DR*01/03, DQ*02/05
	6436	26,3	Body/Head	3,0	Male	Caucasian	30,3	9,0	<0,02	IA-2A+ mIAA+	A*24/29, B*39/45, DR*04/13, DQ*08/06
	6204	28,0	Body	21,0	Male	Caucasian	23	7,2	<0.05	GADA+ mIAA+	A*24/24, B*35/39, DR*07/08, DQ*02/04
	6405	29,1	Head	0,6	Female	Hispanic	42,5	7,0	1,84	GADA+ IA-2A+ ZnT8A+	A*30/31 B*18/61 DR*04/17 DQ*02/08
T2D	6127	44,8	Head	10,0	Female	Caucasian	30,4		0,08	mIAA+	A*01/02, B*08/56, DR*04/17, DQ*02/08
	6133	45,8	Head	20,0	Female	Caucasian	40,2		0,84	Negative	A*02/02, B*03/37, DR*60/10, DQ*15/05
	6124	62,3	Head	3,0	Male	Caucasian	33,7		2,85	Negative	A*29/29, B*27/45, DR*04/15, DQ*06/07

Table S2 | nPOD donor pancreas hormone content, weight and histopathology information. Donor information is sorted by Case-IDs. Information per donor is on isles present with (Ins+) and without (Ins-) insulin, and insulitis (yes/no); fractional insulin area (FIA; insulin area of total area as average from each region; %), fractional glucagon area (FGA, glucagon area of total area as average from each region; %), fractional areas for insulin (INS) and glucagon (GCG) content per pancreatic regions head (PH), body (PB), and tail (PT) (%); total and regional pancreas weight (grams), and histopathology information. Missing data did not pass quality check or is not available.

Condition	Case-ID	INS+	INS-	Insulitis status	FIA	FGA	INS PH	INS PB	INS PT	GCG PH	GCG PB	GCG PT	Pancreatic Weight	Pancreatic weight head	Pancreatic weight body	Pancreatic weight tail	Histopathology
**Control**	6098	Yes	No	No	0,54	0,36	0,51	0,49	0,63	0,34	0,29	0,44	91,1	25,5	28,4	37,2	Insulin+/Glucagon+ normal islets, few with vascular stasis.
	6126	Yes	No	No	0,83	0,43	0,91	0,46	1,11	0,43	0,21	0,66	80,2	18,4	24,9	35,3	Insulin+/Glucagon+ islets, normal. Moderate Ki67 acinar cells and occ. islets.
	6130	Yes	No	No	0,27	0,65	0,16	0,40	0,24	0,47	0,97	0,52	22,2	6,0	8,0	8,2	Insulin+/Glucagon+ normal islets; low Ki67 in acinar and islets. No infiltrates.
	6131	Yes	No	No									108,9	38,4	38,1	28,2	Insulin+/Glucagon+ islets. Occasional high islet Ki67.
	6153	Yes	No	No	0,80	0,30	0,45	1,15		0,34	0,22	0,33	67,8	22,7	23,3	19,9	Insulin+/Glucagon+ islets, numerous. Insulin intensity weak. High Ki67 in islets. Autolysis in head and body regions. No infiltrates islets or exocrine.
	6160	Yes	No	No	0,51	0,59	0,59	0,33	0,60	0,76	0,34	0,68	79,4	20,5	23,1	33,1	Insulin+/Glucagon+ islets present, all sizes. No infiltrates. Low Ki67.
	6227	Yes	No	No	1,21	0,93	1,35	0,94	1,34	0,75	0,75	1,29	60,4	20,9	20,1	19,4	Ins+/Gluc+ islets, no abnormalities observed. Slightly increased acinar Ki67+
	6229	Yes	No	No	3,11	2,14	2,74	2,68	3,91	1,78	1,82	2,81	45,6	14,5	16,2	14,9	Insulin+/Glucagon+ islets, no abnormalities observed. Occasional islet hyperemia.
	6230	Yes	No	No	2,04	1,30	2,09	1,57	2,46	0,81	0,93	2,15	66,3	30,9	20,0	15,3	Ins+/Gluc+ islets, no abnormalities observed
	6232	Yes	No	No	1,79	1,03	1,73	1,18	2,47	0,96	0,73	1,39	49,4	14,0	17,4	18,0	Ins+/Gluc+ islets, numerous. No significant findings. 0-4 Ki67+ cells per islet, some non-insulin, as expected for this age. Low acinar Ki67
	6233	Yes	No	No	2,62	1,03	2,79	2,53	2,54	0,87	1,20	1,01	60,9	17,9	19,2	23,8	Insulin+/Glucagon+ islets, numerous particularly head region. Acinar Ki67 high with moderate islet Ki67 scattered islets. Very mild acute pancreatitis- small foci Ki67+ and/or CD3+ cells, peri-ductular and acinar.
	6331	Yes	No	No									102,7	40,7	31,2	30,8	Ins+/Gluc+ islets, normal numbers and morphologies. Multifocal intralobular duct ruptures due to sludge with acute pancreatitis on background of moderate chronic pancreatitis.
	6374	Yes	No	No									50,5	18,7	16,0	15,7	Ins+/Gluc+ islets, numerous, mostly small to medium sizes. Low islet and acinar cell Ki67+. Mild exocrine infiltrates.
	6412	Yes	No	No									80,8	26,1	25,1	29,7	Ins+/Gluc+ islets, range of sizes including several large (400um) and with irregular outline or elongated. Islet Ki67+ cell numbers high in both endocrine and nonendocrine compartments. Acinar Ki67+ cell numbers variable from low to moderate
	6417	Yes	No	No													Ins+/Gluc+ islets, wide range of densities, sizes and numbers including many single cells, clusters, small islets to several islets >500um. Variable numbers of Ki67+ cells within islets and low numbers in exocrine regions.
**Pregnancy**	6226	Yes	No	No									86,7	27,5	30,3	28,9	Ins+/Gluc+ islets, reduced numbers otherwise no signficiant abnormalities. Moderate autolysis some regions
**AAb+**	6151	Yes	No	No	0,32	0,48	0,66	0,13	0,17	0,62	0,31	0,53	25,0	8,7	6,5	7,1	Insulin+/Glucagon+ islets. No infiltrates observed. Moderate fat infiltrate head region.
	6156	Yes	No	No	0,32	0,57	0,33	0,20	0,43	0,58	0,35	0,78	72,5	18,6	23,3	27,2	Insulin+/Glucagon+ normal islets. Very mild peri-duct and acinar infiltration CD3+.
	6158	Yes	No	No	0,28	0,16	0,21	0,31	0,32	0,01	0,20	0,26	101,9	30,9	26,8	30,5	Insulin+/Glucagon+, some large. Exocrine atrophy- mild. Mild ductular dysplasia. Focal mild chronic pancreatitis.
	6197	Yes	Yes	Yes	2,55	1,34	2,61	1,84	3,19	0,98	1,27	1,78	73,3	14,2	38,8	20,3	Insulin+/Glucagon+ islets, plentiful. Insulitis (rare) with few pseudoatrophic islets. Islet hyperemia. Mild, multifocal chronic pancreatitis. Low Ki67.
	6301	Yes	No	No	2,35	2,50	2,02	1,92	3,12	2,12	1,78	3,62	46,4	17,8	13,0	15,5	Ins+/gluc+ islets, prominent, numerous, some over 500um. Mild acinar atrophy with multiple areas within each section showing acinar cell degranulation. Focal hemorrhages into parenchyma related to trauma.
	6303	Yes	No	No	3,47	1,78	3,51	2,57	4,33	1,51	1,62	2,21	96,2	31,5	27,9	36,8	Ins+/Gluc+ islets, normal numbers and morphologies. High Ki67 islets, acinar, and duct cells. Acute pancreatitis, multifocal, mild to moderate, secondary to pancreatic duct plugging and ruptures; primarily within body but also other regions. Chronic pancreatitis, mild, with periductal fibrosis and active remodeling. Interstitial fibrosis. Lobular necrosis, mild, multifocal. Moderate multifocal hemorrhage all regions.
	6314	Yes	No	No									76,6	21,8	25,3	29,6	Ins+/Gluc+ islets, numerous, several large (200-400um). Islet CD3+ infiltrates not observed. Acinar regions have no significant findings.
	6388	Yes	No	No									101,0	31,7	30,3	39,0	Ins+/Gluc+ islets, range of sizes and morphologies within normal limits. One possible pseudoatrophic islet. Acute and chronic pancreatitis, moderate, multifocal. Pancreatic duct ruptures and/or sludge within lumen. Rupture sites have severe polymorphonuclear infiltrates and mild hemorrhage and fat necrosis (acute pancreatitis). Low Ki67+ cell numbers all regions. Moderate fatty replacement acinar regions and interlobular regions.
	6397	Yes	No	No									70,5	17,3	22,5	30,7	Ins+/Gluc+ islets, range of normal morphologies, sizes, and numbers. Very low acinar Ki67+. Very mild lobular atrophy and perilobular fibrosis. Multifocal mild peripancreatic hemorrhage.
	6400	Yes	No	No									69,6	23,5	21,2	24,9	Ins+/Gluc+ islets, range of sizes and morphologies including very large (500-700um). Variable islet Ki67+ levels, primarily low. Mild global polymorphonuclear exocrine infiltrates. Preliminary- pending screening additional blocks.
	6421	Yes	No	No									28,7	9,7	9,7	9,3	Ins+/Gluc+ islets, within normal range of sizes, morphologies and density between regions. No significant findings other than PanBody 02 shows high Ki67+ cell numbers in main duct epithelium.
	6424	Yes	No	No									111,1	32,9	43,5	34,7	Ins+/Gluc+ islets, normal range of sizes (including > 300um) and morphologies. Moderate increase islet and exocrine Ki67+ cell numbers. Diffuse, mild to moderate increase in exocrine CD3/45+ cell numbers and perivascular or periductal infiltrates. Also compatible with polymorphonuclear infiltrates observed by H&E. Stellate cells are also stained by CD45. Fewer than 3 insulin-negative islets observed so presence of pseudoatrophic islets not supported
	6433	Yes	No	No									82,3	22,1	37,9	22,4	Preliminary- Ins+/Gluc+ islets, wide variation sizes, numbers, and beta-cell proportions. Variable high islet Ki67+ cell numbers, medium to larger sized islets. No pseudoatrophic islets observed to date. Microadenoma PB 04A. PanIN, low grade, multiple. Acinar atrophy with fibrosis.
**T1D**	6063	No	Yes	No	0,05	0,39	0,02	0,06	0,08	0,26	0,42	0,49	11,2	3,9	2,6	2,7	Insulin-/Glucagon+ islets, clusters and single cells, reduced islet numbers. Islets with possible infiltrates (<3 islets/section). Infrequent CD3+ acinar infiltrates.
	6064	No	Yes	No	0,01	0,24	0,01	0,01	0,01	0,13	0,13	0,46	46,2	11,2	16,5	17,1	Insulin-/Glucagon+ islets, reduced frequency and area of islets; acute pancreatitis (PMN); Ki67+ acinar region, ducts, and infiltrating cells
	6087	No	Yes	No	0,01	0,57	0,00	0,04	0,00	0,33	0,43	0,96	58,4	21,8	18,5	17,7	Insulin-/Glucagon+ islets- reduced frequency. Mild, focal CD3+ islet infiltrates.
	6113	Yes	Yes	Yes	0,02	0,44	0,01	0,04	0,01	0,53	0,30	0,50	33,5	11,1	10,7	10,2	Insulin+ (reduced)/Glucagon+ islets. Insulitis. Lobular islet heterogeneity. Islet atrophy with mild acinar atrophy (heterogeneous). Glucagon cells as single cells and small clusters.
	6198	Yes	Yes	Yes	0,30	1,79			0,30			1,79					Insulin+/Glucagon+ islets. Diffuse mild insulitis (increase in CD3+cells/islet rather than focal aggregates). Moderate to high acinar Ki67+, low islet. Mild diffuse chronic pancreatitis. Mild acinar atrophy and ductular proliferation.
	6204	No	Yes	No	0,01	2,75	0,02	0,01			2,41	3,08	55,0	25,8	19,0	10,2	Insulin-/Glucagon+ islets. High duct and exocrine Ki67. Small duct plugging with rupture. Hemorrhage and acinar degeneration/necrosis with other areas of mild exocrine atrophy. Acute and chronic pancreatitis.
	6209	Yes	Yes	Yes	0,17	2,17	0,14	0,22	0,14	1,07	1,91	3,54	12,5	4,7	4,5	3,3	Insulin+ (reduced)/Glucagon+ islets with insulitis. Low Ki67 in all cell types. No pancreatitis. Moderate autolysis.
	6211	Yes	Yes	No	0,09	1,97	0,04	0,06	0,18	0,93	1,68	3,30	33,0	11,0	12,0	10,0	Ins+(reduced)/Gluc+ islets, numerous. Alpha cells in ducts appear more frequent than expected. Very mild chronic pancreatitis- infrequent, lobular
	6228	Yes	Yes	Yes	0,34	1,45	0,11	0,50	0,40	1,10	1,57	1,68	30,4	11,2	10,8	8,5	Insulin+/Glucagon+ Islets of various morphologies including normal appearing, fibrotic, degenerating, and with insulitis with infiltrate that includes eosinophils. Some pseudoatrophic (glucagon+ only) islets. Very mild, multifocal exocrine atrophy. Mild, multifocal mononuclear infiltrate with focally moderate (chronic pancreatitis).Increased exocrine and endocrine Ki67+. Eosinophils observed within islets and in exocrine areas in increased numbers.
	6243	Yes	Yes	Yes	0,50	2,21	0,48	0,38	0,65	1,55	1,96	3,10	29,4	10,4	10,5	8,5	Ins+/Gluc+ islets and single beta-cells (reduced numbers but present throughout all blocks), glucagon+ only islets (pseudoatrophic). Insulitis present particularly PanTail. Low normal Ki67 islets and acinar cells. Variable mild fatty exocrine infiltration. Hydropic degeneration in scattered islets within most regions. Diffuse moderate CD3+ exocrine infiltrates and Ki67+ immune cells.
	6337	Yes	Yes	No									26,8	7,8	10,2	8,8	Ins+ (rare)/Gluc+ islets, reduced numbers and sizes. Moderate acinar atrophy. Minimal exocrine CD3+ infiltrates and low Ki67+ acinar cells. Severe focal duodenal ulceration- denuded epithelium, acute mixed infiltrate with hemorrhage, necrosis, suppuration with extension into muscular layers.
	6360	No	Yes	No									10,3	3,2	3,5	3,6	Ins-/Gluc+ islets, reduced numbers, mostly small. Somatostatin may be slightly increased (# cells/islet). Low Ki67 acinar cells and ducts. Mild CD3+ exocrine infiltrates head and body regions. Mild acinar atrophy and periductal fibrosis.
	6362	Yes	Yes	Yes	0,30	1,37	0,20	0,20	0,47	0,84	1,06	2,22	53,3	13,1	17,2	23,0	Ins+/Gluc+ islets, moderate reduction numbers of Ins+ islets. Many islets with abnormal morphologies (large, fusing smaller islets, irregular outlines, fibrosis) in contrast to normal spherical/oblong islet morphologies. Insulitis in most Ins+ islets and small number of Ins- islets. Islet nuclear pleomorphism with variable hydropic degeneration. Adipose cells are also associated with islets. Generally low islet Ki67+ but occasional islet with high Ki67+ in non-beta cells. Moderate to high acinar Ki67+ cells. Mild to moderate acinar CD3+ infiltrates and variable fatty infiltration. Moderate exocrine atrophy with variable intralobular fibrosis. Amyloid in rare islets, PanTail.
	6380	Yes	Yes	Yes									34,3	10,5	14,3	9,4	Ins+ (reduced)/Gluc+ islets, mostly small and reduced numbers. Pseudoatrophic islets (insulin-negative) are highly variable with expected heterogeneity in lobular distribution. Variable moderate Ki67+ cells in all exocrine regions with rare islet observed with moderate Ki67+ cell numbers. Possible insulitis seen in both insulin+ and insulin- islets with rarely more than 6 CD3+ cells diffusely associated with islet periphery. CD45+ cells diffusely increased exocrine regions with mild to moderate increase in polymorphonuclear cells, particularly eosinophils. Most infiltrates appear peri-vascular. No fibrosis or exocrine atrophy.
	6405	Yes	Yes	Yes									94,3	34,3	29,8	30,3	Ins+/Gluc+ (majority) with normal sizes and morphologies. Ins-/Gluc+ islets as scattered small islets or within certain lobules. Insulitis present all regions. Variable peri-islet fibrosis. Acute pancreatitis (PH 01A). Lobules with moderate acinar Ki67+ cell numbers. Fatty infiltration highly variable between lobules given block. Multiple small foci of lobular fibrosis with low grade PanIN. Multifocal squamous metaplasia in small intralobular ducts.
	6436	Yes	Yes	No									57,1	20,1	17,2	19,8	Ins+/Gluc+ islets with hypertrophy and Ins+ clusters and single BC. Insulitis at Ins- and Ins- islets. Islet hypertrophy and irregular morphologies. Amyloid in at least 1 islet (PT). Peri-pancreatic trauma with acute pancreatitis PB 02-04 with evidence of low grade PanIN in regions with interstitial fibrosis (mild chronic pancreatitis PH 04B).
**T2D**	6124	Yes	No	No	0,28	0,63	0,28	0,31	0,25	0,39	0,92	0,59	103,9	36,3	22,0	38,1	Insulin+/Glucagon+, normal islet density. Hypertrophied islets. Amyloid. Acinar atrophy- moderate to severe. Peri-islet fibrosis.
	6127	Yes	Yes	No	0,04	0,82	0,07	0,03	0,03	1,25	0,58	0,63	58,9	21,2	16,7	15,5	Insulin+ (reduced)/Glucagon+ islets. Mild adiposity exocrine regions. Low Ki67. Pseudoatrophic islets and severe amyloid confirmed with additional IHC and Congo Red stains as well as infiltrating CD3+ cells in islets, with and without insulin and amyloid.
	6133	Yes	No	No	0,17	0,19	0,26	0,09	0,15	0,06	0,15	0,37	70,4	31,1	17,2	19,0	Insulin+/Glucagon+ islets, many w/ amyloid. Multifocal duct epithelium Ki67+. Mild, interstitial CD3+ infiltrate. Duct sludge and acute pancreatitis. Severe exocrine atrophy and arteriosclerosis. Fatty replacement of exocrine tissues with multifocal fat necrosis.
