# Supplementary material for: Large-scale electron microscopy database for human type 1 diabetes
Source: Nat Commun. 2020 May 18;11:2475. doi: 10.1038/s41467-020-16287-5 (PMC7235089; doi:10.1038/s41467-020-16287-5)
Supplement: Supplementary file 3 — Description of Additional Supplementary Files [file 41467_2020_16287_MOESM3_ESM.pdf]

Description of Additional Supplementary Files

File Name: Supplementary Data 1

Description: Supplementary dataset file: Extended EDX data and information on diabetes-related donor parameters are available in the supplementary dataset file.
