# Supplementary material for: Large-scale electron microscopy database for human type 1 diabetes
Source: Nat Commun. 2020 May 18;11:2475. doi: 10.1038/s41467-020-16287-5 (PMC7235089; doi:10.1038/s41467-020-16287-5)
Supplement: Supplementary file 4 — Reporting Summary [file 41467_2020_16287_MOESM4_ESM.pdf]

nature research

Corresponding author(s): BNG Giepmans

Last updated by author(s): Apr 03, 2020

Reporting Summary

Nature Research wishes to improve the reproducibility of the work that we publish. This form provides structure for consistency and transparency in reporting. For further information on Nature Research policies, see Authors & Referees and the Editorial Policy Checklist.

[illegible]tatistics

[illegible]or all statistical analyses, confirm that the following items are present in the figure legend, table legend, main text, or Methods section.

n/a	Confirmed	
☐	☒	The exact sample size (*n*) for each experimental group/condition, given as a discrete number and unit of measurement
☐	☒	A statement on whether measurements were taken from distinct samples or whether the same sample was measured repeatedly
☐	☒	The statistical test(s) used AND whether they are one- or two-sided<br>*Only common tests should be described solely by name; describe more complex techniques in the Methods section.*
☐	☒	A description of all covariates tested
☒	☐	A description of any assumptions or corrections, such as tests of normality and adjustment for multiple comparisons
☐	☒	A full description of the statistical parameters including central tendency (e.g. means) or other basic estimates (e.g. regression coefficient) AND variation (e.g. standard deviation) or associated estimates of uncertainty (e.g. confidence intervals)
☐	☒	For null hypothesis testing, the test statistic (e.g. *F*, *t*, *r*) with confidence intervals, effect sizes, degrees of freedom and *P* value noted<br>*Give P values as exact values whenever suitable.*
☒	☐	For Bayesian analysis, information on the choice of priors and Markov chain Monte Carlo settings
☒	☐	For hierarchical and complex designs, identification of the appropriate level for tests and full reporting of outcomes
☒	☐	Estimates of effect sizes (e.g. Cohen's *d*, Pearson's *r*), indicating how they were calculated

Our web collection on statistics for biologists contains articles on many of the points above.

Software and code

Policy information about availability of computer code

[illegible]ata collection	Large-scale EM: Zeiss supra55 Scanning EM with Fibics external scan generator ATLAS 5. EDX data with AZtecEnergy software
[illegible]ata analysis	ATLAS 5 and ATLAS Browser-based viewer, Adobe Photoshop 19.1.5, IBM SPSS Statistics V25

[illegible]manuscripts utilizing custom algorithms or software that are central to the research but not yet described in published literature, software must be made available to editors/reviewers. We strongly encourage code deposition in a community repository (e.g. GitHub). See the Nature Research guidelines for submitting code & software for further information.

[illegible]ata

Policy information about availability of data

All manuscripts must include a data availability statement. This statement should provide the following information, where applicable:

- Accession codes, unique identifiers, or web links for publicly available datasets
- A list of figures that have associated raw data
- A description of any restrictions on data availability

All data is open access at www.nanotomy.org (www.nanotomy.org./OA/nPOD) and have a unique accession code matching the donor number, like many other datsets published via the NPG. Currently, other repositories downscale the data, but we aim to have this accessible in the near future in these repositories (2020/2021). A collaborative effort is ongoing.

Field-specific reporting

Please select the one below that is the best fit for your research. If you are not sure, read the appropriate sections before making your selection.

☒ Life sciences ☐ Behavioural & social sciences ☐ Ecological, evolutionary & environmental sciences

For a reference copy of the document with all sections, see nature.com/documents/nr-reporting-summary-flat.pdf

Life sciences study design

All studies must disclose on these points even when the disclosure is negative.

Sample size	This study was completely dependent on nPOD pancreas donor material availability. A power analysis indicated that a minimum of 13 donors per group were needed for statements about different immune cell and intermediate cell prevalence.
Data exclusions	Data of one donor was excluded based on not passing EM quality control on ultrastructural morphology preservation.
eplication	All material is unique therefore also each observation, but groups were formed to non-diabetic controls (n=16), autoantibody-positive donors without type 1 diabetes (n=13), and donors with type 1 diabetes (n=16). Each dataset was analyzed for parameters of interest.
Randomization	Groups were based on donor type, non-diabetic controls, autoantibody-positive donors without type 1 diabetes, and donors with type 1 diabetes, which precluded any randomization.
inding	N/A, donor status was essential for group assignment

Reporting for specific materials, systems and methods

We require information from authors about some types of materials, experimental systems and methods used in many studies. Here, indicate whether each material, system or method listed is relevant to your study. If you are not sure if a list item applies to your research, read the appropriate section before selecting a response.

Materials & experimental systems

n/a	Involved in the study	
☒	☐	Antibodies
☒	☐	Eukaryotic cell lines
☒	☐	Palaeontology
☒	☐	Animals and other organisms
☐	☒	Human research participants
☒	☐	Clinical data

Methods

n/a	Involved in the study	
☒	☐	ChIP-seq
☒	☐	Flow cytometry
☒	☐	MRI-based neuroimaging

Human research participants

Policy information about studies involving human research participants Population characteristics

Potentially any deceased persons in the US. All Co-variates can be obtained from Supplementary table 1

ecruitment

We aim to obtain pancreata and nanotomy maps of all nPOD type 1 diabetes donores, as well as autoantibody positive without diabetes donors and aim to match them with control donors (age, sex, BMI, etc). Note that the project is ongoing and the biobank is therefore growing.

Ethics oversight

University of Florida, FL, USA and confirmed by the Medical Ethical Committee of UMC Groningen, Netherlands.

Note that full information on the approval of the study protocol must also be provided in the manuscript.
